# Supplementary material for: Genomic dissection and mutation-specific target discovery for breast cancer PIK3CA hotspot mutations
Source: BMC Genomics. 2024 May 27;25:519. doi: 10.1186/s12864-024-10368-1 (PMC11129441; doi:10.1186/s12864-024-10368-1)
Supplement: Supplementary file 1 — Supplementary Material 1. [file 12864_2024_10368_MOESM1_ESM.pdf]

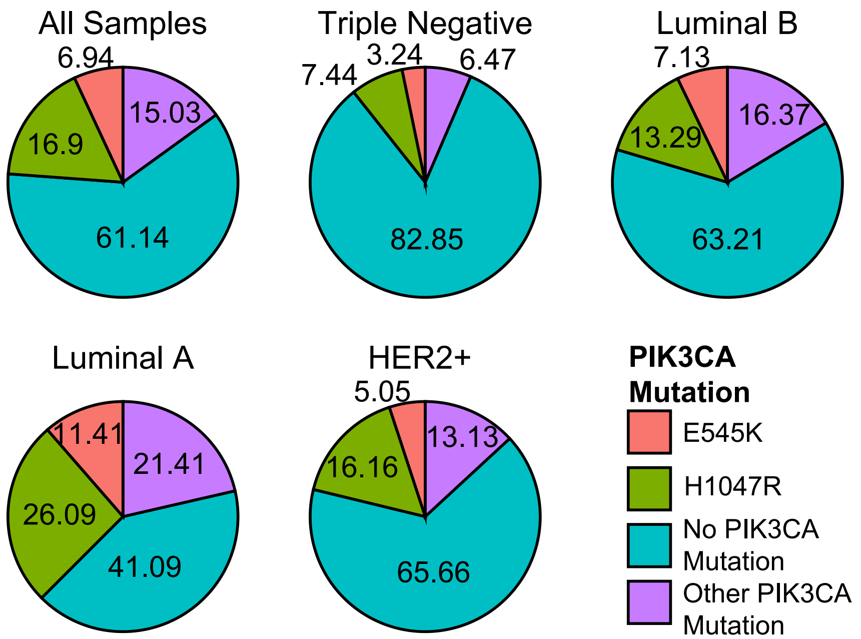

Figure S1.
Pie charts showing the proportion of *PIK3CA* mutations in the METABRIC study from cbioPortal stratified by breast cancer subtype.

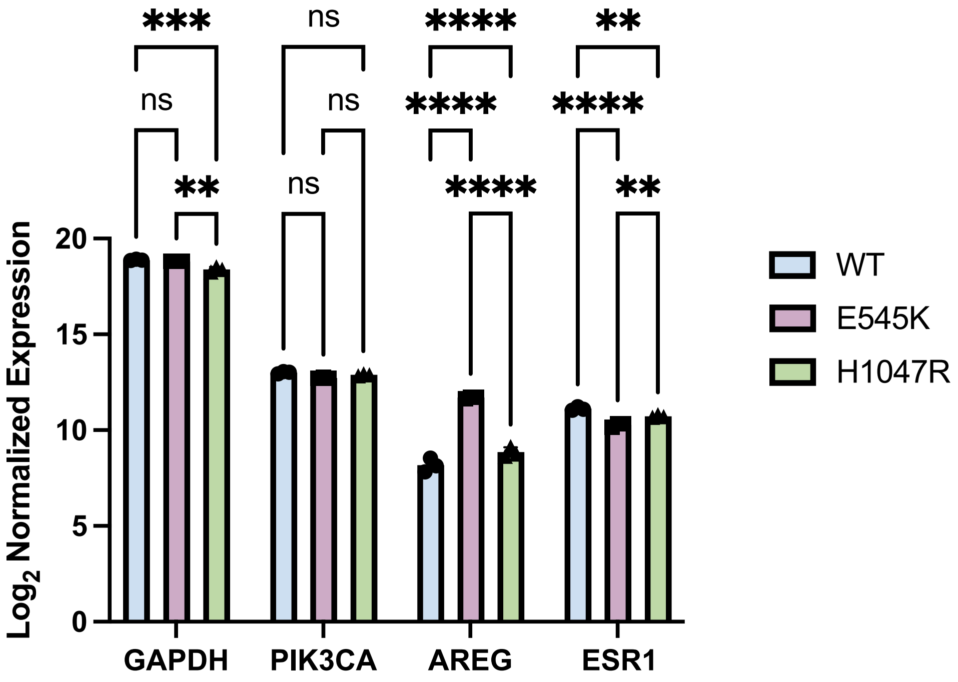

Figure S2.

Bar chart showing log normalized counts expression of key genes from RNA-seq of cell lines in the MCF-10A isogenic cell line model. The significance tests shown are based on a two-way ANOVA and a post-hoc Tukey test. These are not representative of differential expression analyses shown in other figures; * = p-value 0.05-0.0332, ** = p-value .0332-0.0021, *** = p-value 0.0021-0.0002, ****= p-value <0.0002.

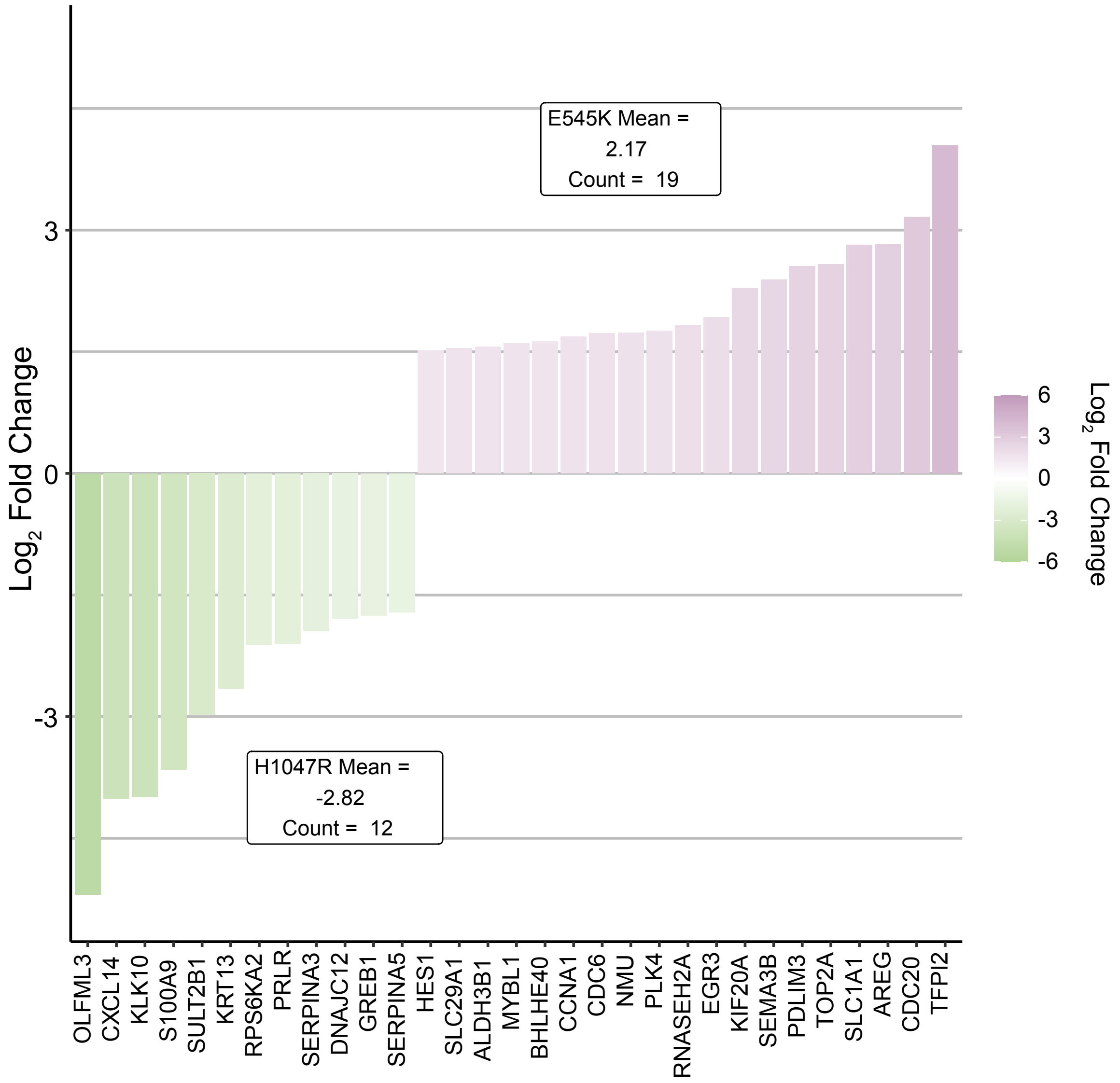

Figure S3.
Bar plot showing the Log_2 Fold Change of DEGs within the estrogen response late and estrogen response early hallmark pathways.

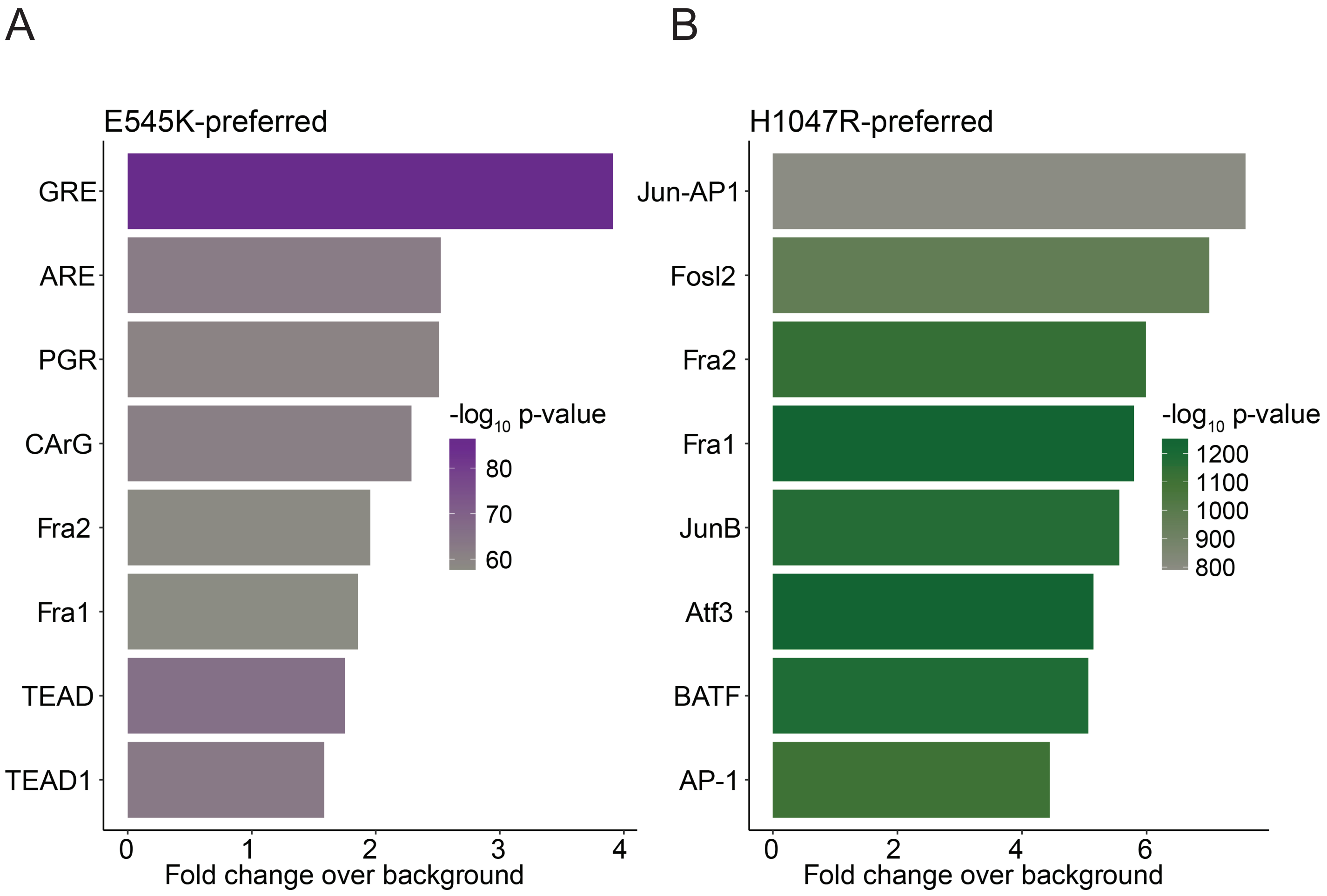

Figure S4.
Bar plots showing the results of HOMER TF motif enrichment analysis within the (A) E545K-preferred and (B) H1047R-preferred cluster regions.

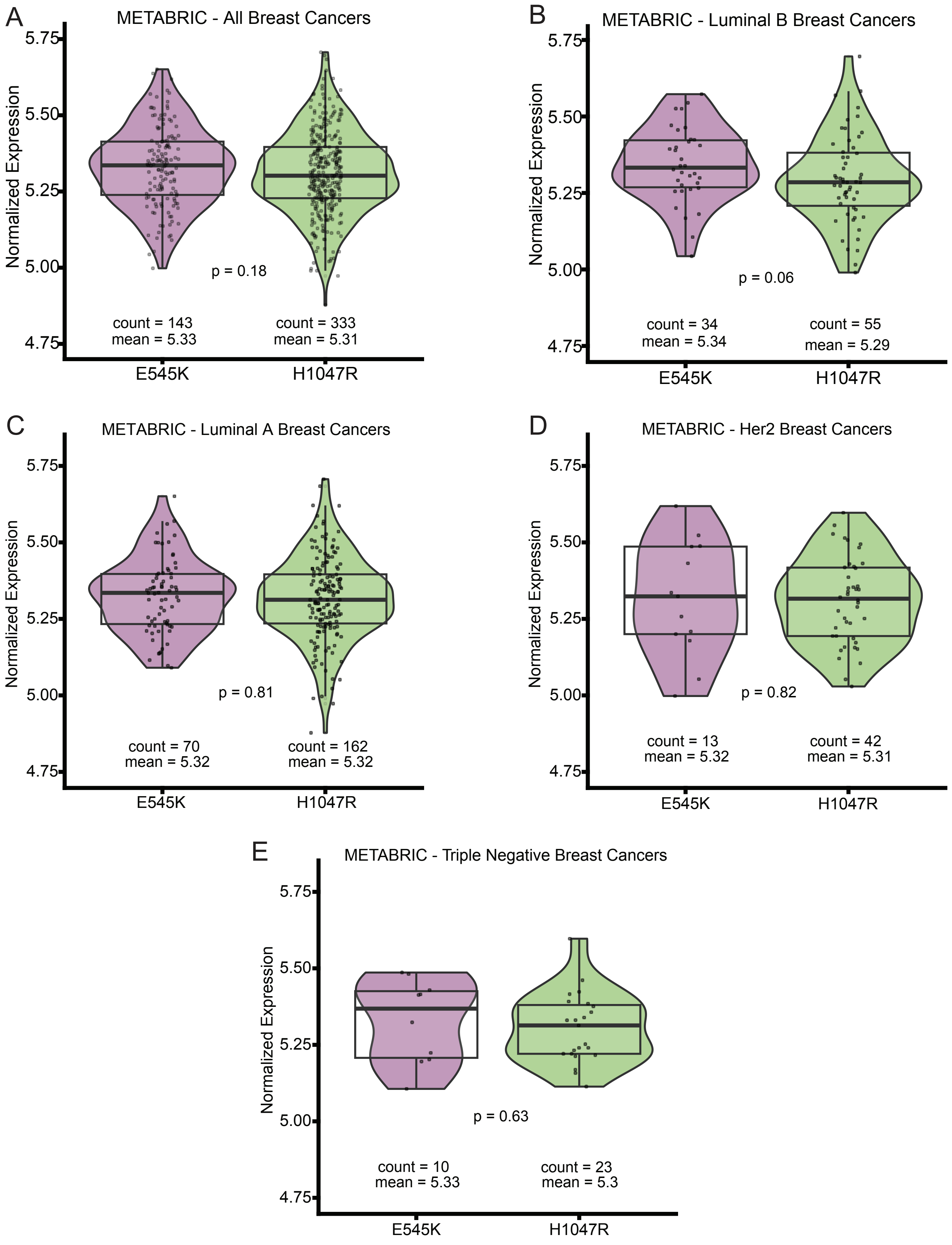

Figure S5.
Violin plots showing the expression of AREG from METABRIC samples with either PIK3CA hotspot mutation. (A) AREG expression in all samples. (B) AREG expression from samples specifically of the luminal B subtype. (C) AREG expression in samples with the luminal A subtype. (D) Expression in samples with the Her2 subtype. (E) Expression in samples of the Triple Negative Subtype. *P*-values were calculated using the Wilcoxon test.

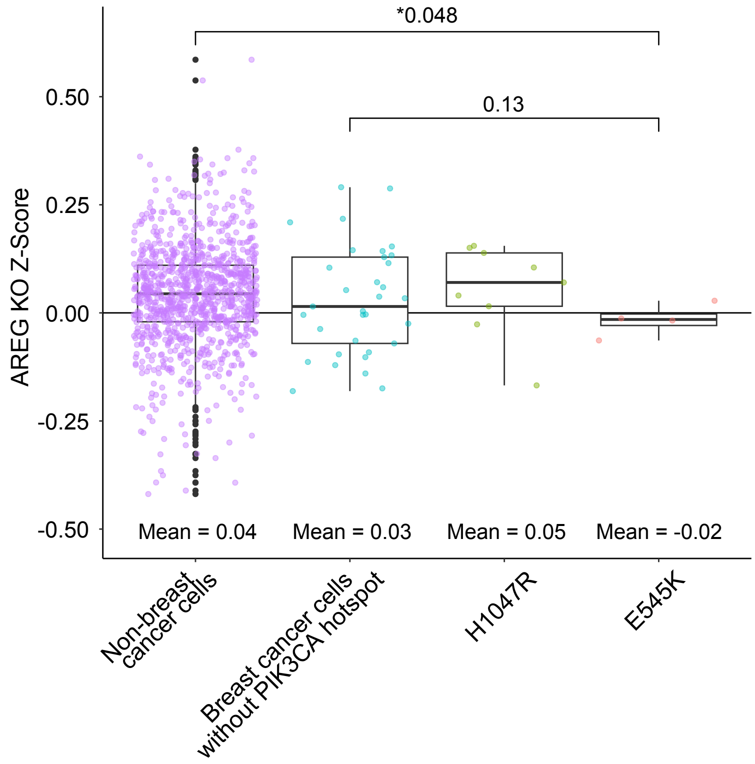

Figure S6.

Box and whisker plots showing the Z-score of AREG KO in all cell lines from the DepMap study stratified by cancer type and *PIK3CA* mutation. *P*-values were calculated using a nonparametric t-test.

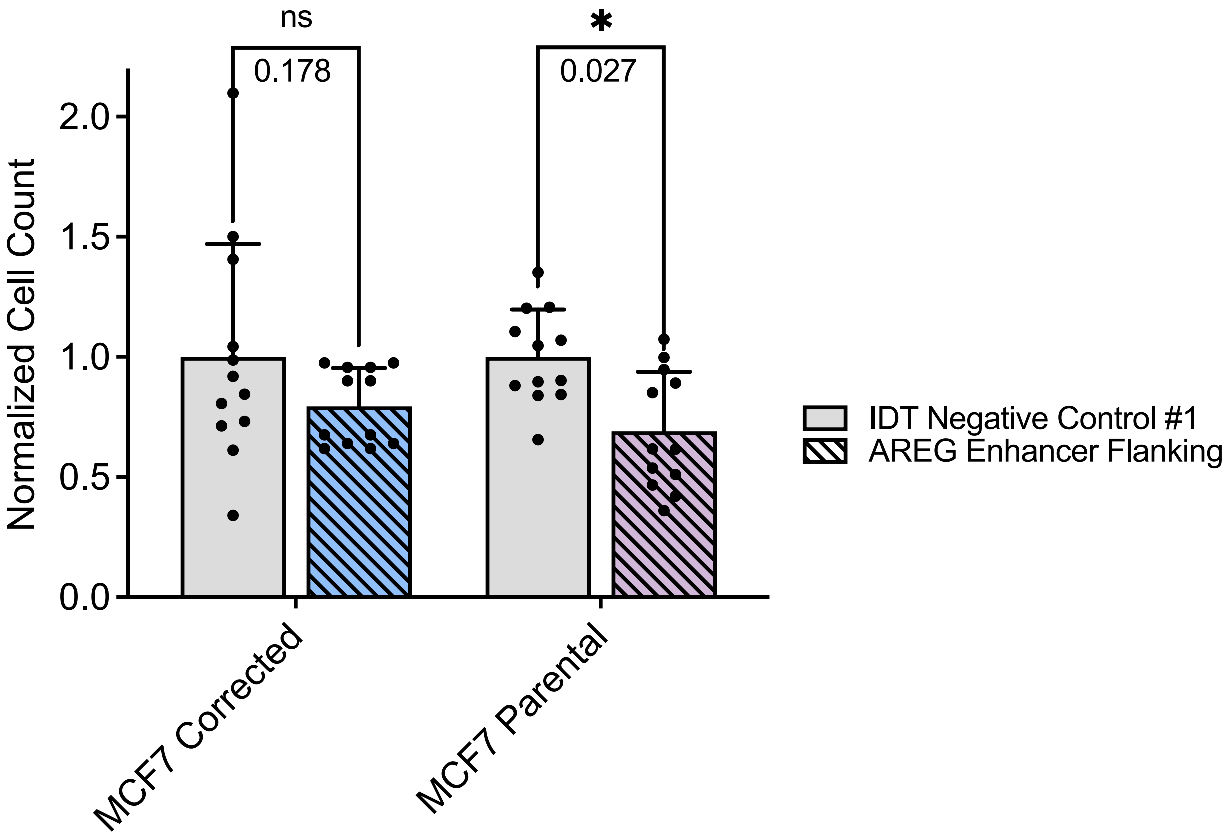

Figure S7.
Bar plot of cell counts following CRISPR-mediated deletion of the putative AREG enhancer. Significance was calculated using ANOVA with a post-hoc Šidák's test with a significance threshold of 0.05.

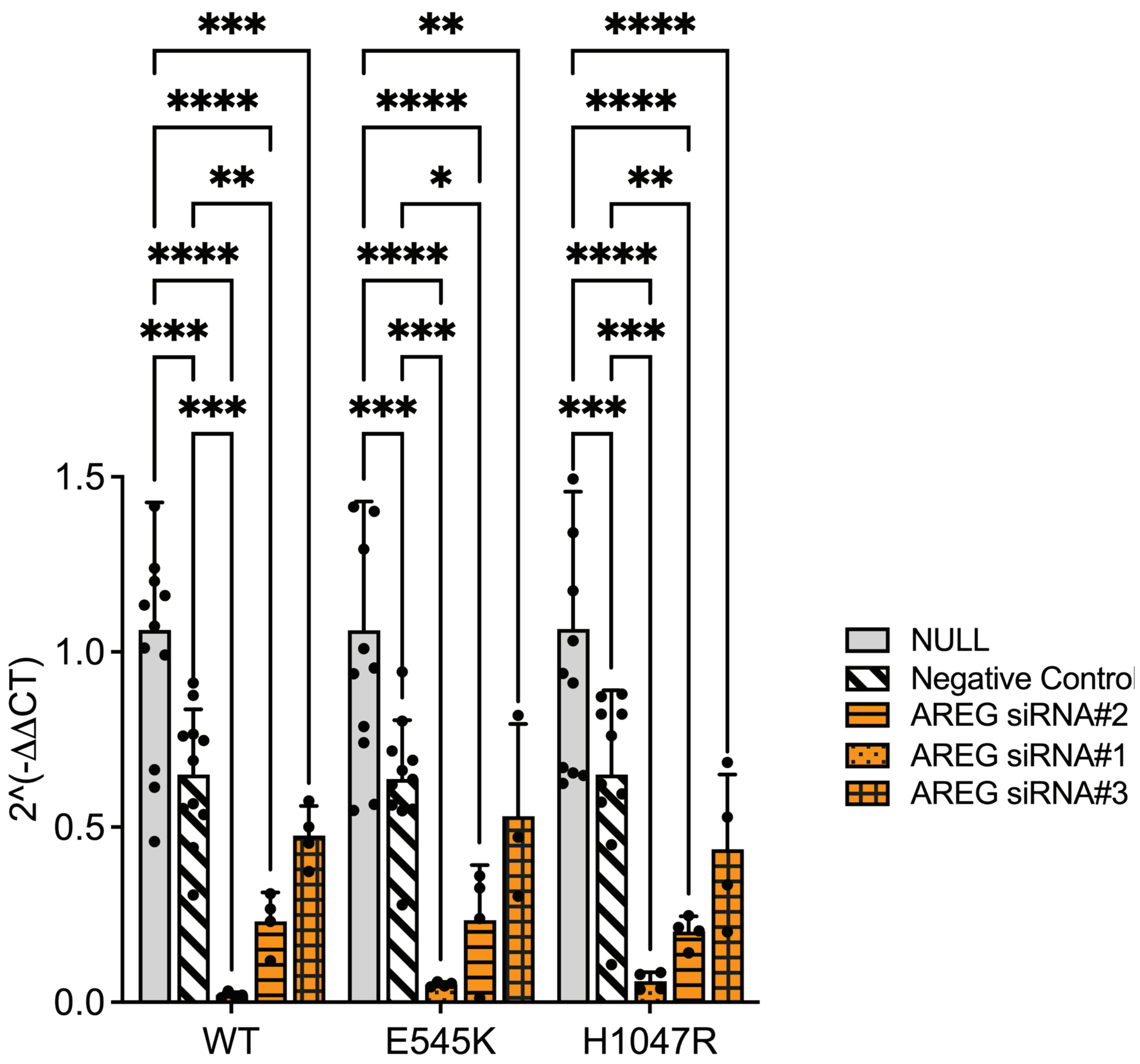

Figure S8.
Bar plots showing the differences in the expression of AREG relative to ACTB following siRNA-mediated inhibition of AREG with each of the three AREG-targeting siRNA molecules listed in Table S5. Outliers were removed using the ROUT method with a Q threshold of 1%. Significance calculated using an ANOVA with a post-hoc Fisher's LSD test; * = p-value 0.05-0.0332, ** = p-value .0332-0.0021, *** = p-value 0.0021-0.0002, ****= p-value <0.0002.

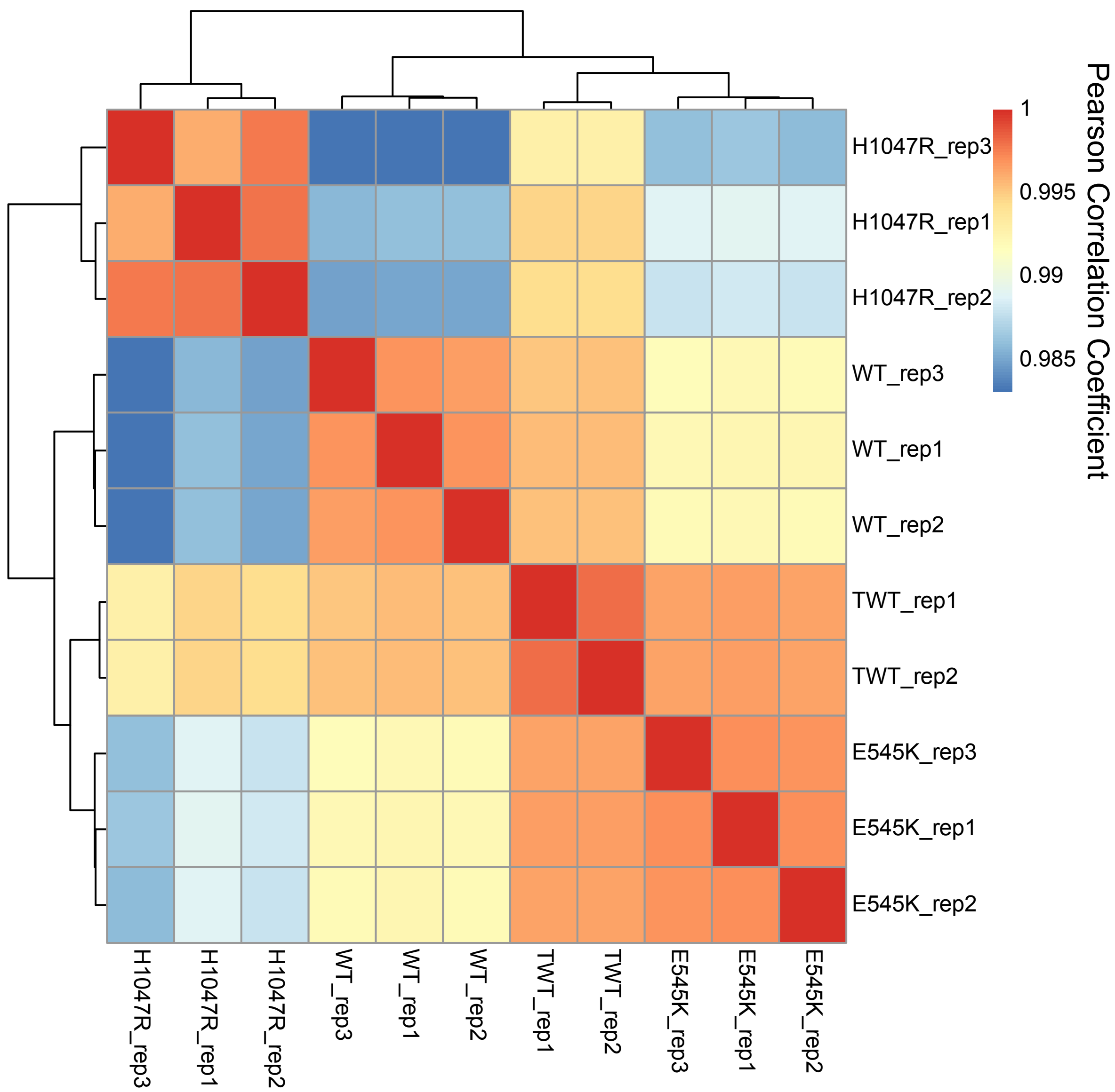

Figure S9.
Heatmap plots the pearson correlation coefficient across batch corrected RNA-seq gene counts. Samples are clustered by hierarchical clustering. TWT represents targeted WT cells from Dalton et al. 2019.

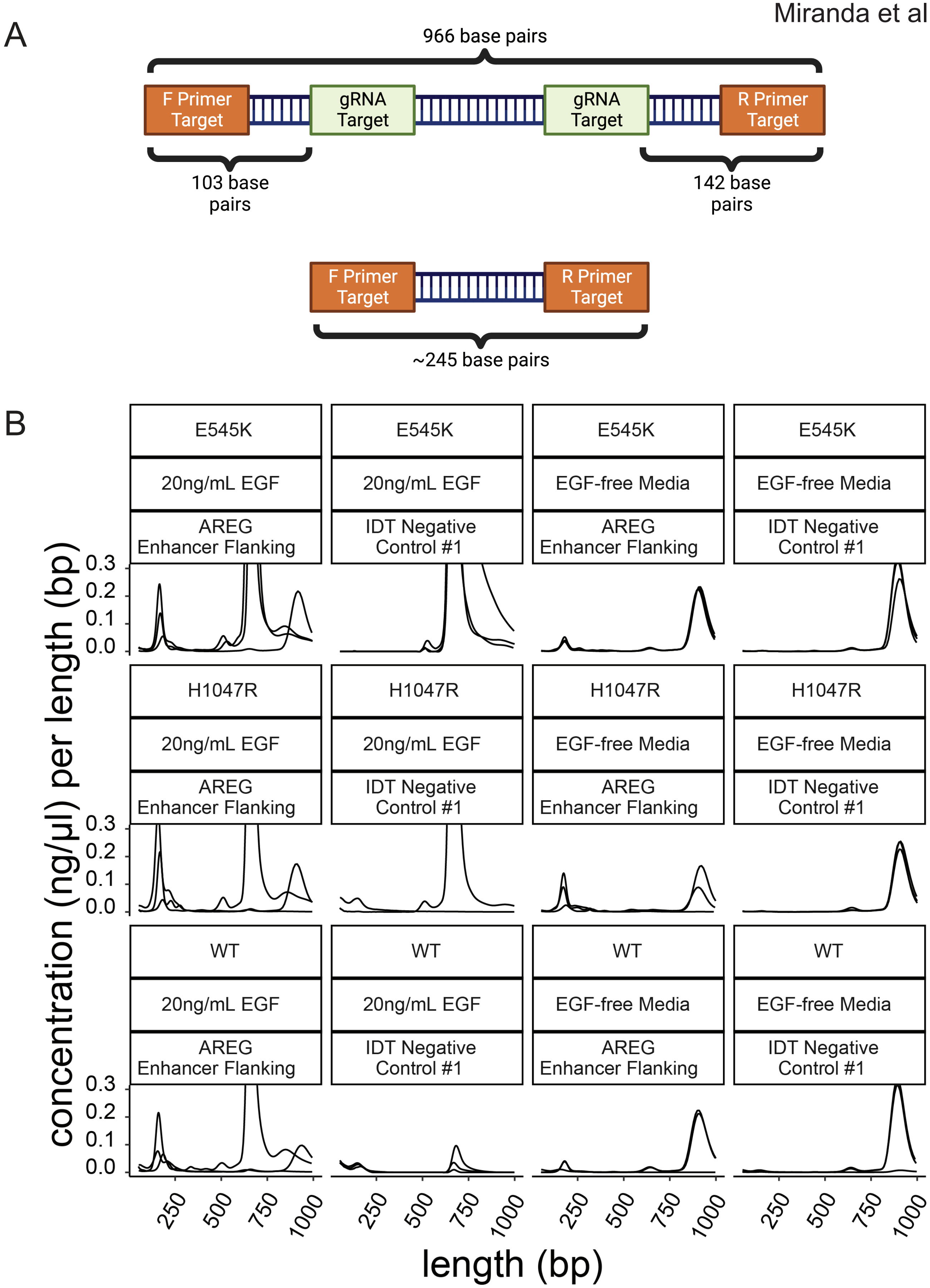

Figure S10.
Validation of enhancer region deletion. (A) Schematic showing the predicted PCR product sizes based on the deletion of the targeted region. A PCR product of 966 bp corresponds to the unmodified enhancer region. The PCR product of ~245bp signifies a deleted enhancer. (B) Reduced electropherograms from D5000 tapestation from 3 replicates of all cell lines and all media conditions. The presence of the ~250bp band is more prevalent in cells that received the AREG enhancer flanking guides compared to the negative control.
